# Decoding COVID-19 pneumonia: comparison of deep learning and radiomics CT image signatures

**DOI:** 10.1007/s00259-020-05075-4

**Published:** 2020-10-23

**Authors:** Hongmei Wang, Lu Wang, Edward H. Lee, Jimmy Zheng, Wei Zhang, Safwan Halabi, Chunlei Liu, Kexue Deng, Jiangdian Song, Kristen W. Yeom

**Affiliations:** 1 Department of Radiology, The First Affiliated Hospital of University of Science and Technology of China (USTC), Division of Life Sciences and Medicine, USTC, Hefei, 230036 Anhui China; 2 School of Medical Informatics, China Medical University, Shenyang, 110122 Liaoning China; 3 Department of Radiology, School of Medicine Stanford University, 725 Welch Rd MC 5654, Palo Alto, CA 94305 USA; 4 Department of Radiology, The Lu’an Affiliated Hospital, Anhui Medical University, Luan, 237000 Anhui China; 5 Department of Electrical Engineering and Computer Sciences, University of California, Berkeley, CA 94720 USA; 6 Helen Wills Neuroscience Institute, University of California, Berkeley, CA 94720 USA; 7 College of Medical Informatics, China Medical University, Shenyang, 110122 Liaoning China; 8 Department of Radiology, School of Medicine Stanford University, 1201 Welch Rd Lucas Center PS055, Stanford, CA 94305 USA

**Keywords:** Coronavirus disease 2019 pneumonia, CT chest, Machine learning, AI interpretability, Explainable AI

## Abstract

**Purpose:**

High-dimensional image features that underlie COVID-19 pneumonia remain opaque. We aim to compare feature engineering and deep learning methods to gain insights into the image features that drive CT-based for COVID-19 pneumonia prediction, and uncover CT image features significant for COVID-19 pneumonia from deep learning and radiomics framework.

**Methods:**

A total of 266 patients with COVID-19 and other viral pneumonia with clinical symptoms and CT signs similar to that of COVID-19 during the outbreak were retrospectively collected from three hospitals in China and the USA. All the pneumonia lesions on CT images were manually delineated by four radiologists. One hundred eighty-four patients (*n* = 93 COVID-19 positive; *n* = 91 COVID-19 negative; 24,216 pneumonia lesions from 12,001 CT image slices) from two hospitals from China served as discovery cohort for model development. Thirty-two patients (17 COVID-19 positive, 15 COVID-19 negative; 7883 pneumonia lesions from 3799 CT image slices) from a US hospital served as external validation cohort. A bi-directional adversarial network-based framework and PyRadiomics package were used to extract deep learning and radiomics features, respectively. Linear and Lasso classifiers were used to develop models predictive of COVID-19 versus non-COVID-19 viral pneumonia.

**Results:**

120-dimensional deep learning image features and 120-dimensional radiomics features were extracted. Linear and Lasso classifiers identified 32 high-dimensional deep learning image features and 4 radiomics features associated with COVID-19 pneumonia diagnosis (*P* < 0.0001). Both models achieved sensitivity > 73% and specificity > 75% on external validation cohort with slight superior performance for radiomics Lasso classifier. Human expert diagnostic performance improved (increase by 16.5% and 11.6% in sensitivity and specificity, respectively) when using a combined deep learning-radiomics model.

**Conclusions:**

We uncover specific deep learning and radiomics features to add insight into interpretability of machine learning algorithms and compare deep learning and radiomics models for COVID-19 pneumonia that might serve to augment human diagnostic performance.

**Electronic supplementary material:**

The online version of this article (10.1007/s00259-020-05075-4) contains supplementary material, which is available to authorized users.

## Introduction

The coronavirus disease (COVID-19) pandemic has caused more than 10.1 million infections and 503,000 deaths worldwide as of June 30, 2020 [1]. The virus nucleic acid real-time reverse transcriptase chain reaction (RT-PCR) test is the current recommended method for COVID-19 diagnosis [2, 3]. However, with the rapid increase in the number of infections, RT-PCR tests may be fallible depending viral load or sampling techniques and may vary in its availability across global regions. Multiple studies have shown utility for chest CT for diagnosis of COVID-19 [4–6], including reports of diagnostic accuracy of chest CT > 80% using deep learning (DL) approaches [7, 8].

While these studies often report classification performance, i.e., positive or negative for COVID-19 pneumonia [8, 9], investigations on specific high-dimensional features unique to COVID-19 pneumonia compared to other similar-appearing lung diseases remain relatively unexplored. Furthermore, there is paucity of research that directly compares predictive performance of feature engineering (e.g., radiomics), deep learning, and other machine learning approaches [10]. While it is generally accepted that deep learning performs robustly with large datasets for image discrimination, its performance compared to radiomics is not closely examined, a relevant topic in medicine where sample sizes are much smaller than non-medical image datasets, such as ImageNet. The types of machine learning approaches that most optimally augment clinician performance also remain unknown.

A recently described neural network using large-scale bi-directional adversarial network (BigBiGAN) has shown potential for an end-to-end COVID-19 pneumonia diagnosis [11]. In this network, CT chest images are taken as the input and high-dimensional semantic features representing specific characteristics of each image are produced based on the modules of image encoding, image generation, and image discrimination. A recent study has shown a potential utility of this method for distinguishing COVID-19 pneumonia from other viral pneumonias [12].

In this study, we aim to uncover image features of COVID-19 lung disease and further compare radiomics versus deep learning model performance using chest CT. Specifically, we target COVID-19 and non-COVID-19 viral pneumonia of patients who presented with similar clinical symptoms and CT chest findings and compare the performance of deep learning features, radiomics features, and combined approaches for COVID-19 pneumonia diagnosis.

## Material and methods

### Study cohort

The inclusion criteria of this retrospective study were patients with symptoms suspicious for COVID-19 and diagnosed with COVID-19 or non-COVID-19 viral pneumonia during the COVID-19 outbreak; patients obtained CT chest with or without contrast at time of diagnosis; patients obtained RT-PCR tests (based on samples of bronchoalveolar lavages, endotracheal aspirates, nasopharyngeal swabs, or oropharyngeal swabs) to determine COVID-19 status. Only those patients who tested positive or negative on at least *two* RT-PCR tests were included. Patients who were confirmed to have PCR-confirmed COVID-19 pneumonia with underlying lung diseases (e.g., lung cancer) were included. Lesion segmentation was performed only on lung lesions suspicious for pneumonia, excluding known sites of lung cancer or other chronic lung lesions. For non-COVID-19 pneumonia patients, we included patients clinically suspected to have viral source of infection. Tuberculosis, fungal, or bacterial pneumonia patients were excluded to examine specific image features of non-COVID-19 viral pneumonia. This retrospective study was approved by the institutional review board of University of Science and Technology of China (IRB no.2020-P-038) and Stanford University (IRB no.51059), with waiver of informed consent or assent.

### Chest CT technique and image annotations

Chest CT was performed at slice thickness range 1.25–5 mm (NeuViz 64 or 128, Neusoft, Shenyang, China) with or without contrast. CT parameters of the external dataset were 1–3 mm slice thickness with or without contrast (LightSpeed VCT and Revolution, GE Healthcare, Milwaukee, WI; Aquilion, Toshiba Medical Systems, Otawara, Japan; SOMATOM, Siemens, Erlangen, Germany).

Four blinded attending radiologists in China (> 5 years’ experience) independently segmented the boundary of all lung lesions slice-by-slice using ITK-Snap software (v.3.6.0). Detailed segmentation procedure is presented in Supplementary Figure S1. All segmentations underwent quality control for proper annotation by an expert chest radiologist (> 10 years’ experience). Images were not segmented if the radiologists did not detect lung lesions.

### Feature extraction and model development

Open access Google Colab platform [13] and PyRadiomics [14] were used for DL and radiomics feature extraction, respectively. CT data from two hospitals from China were randomly divided into a training, validation, and test datasets (80:10:10), and the data was processed on servers in China. Dataset from a US hospital served as an external evaluation and processed on servers in the USA.

#### High-dimensional deep learning features

We used a BigBiGAN-based architecture to train and extract high-dimensional deep learning features of COVID-19 versus non-COVID-19 pneumonia lesions. Two different data inputs were used: (1) original CT with segmentation masks, where the pixels of the pneumonia lesions were retained as the original CT intensity and the pixels outside were set to zero (Supplementary Figure S2); (2) CT images of the whole lung without segmentation mask. The batch size and epoch of the BigBiGAN training were set as 20 and 200, respectively. The 120-dimensional deep learning features were extracted by the encoder module of BigBiGAN when the loss was minimum in the last training epoch, which then served as input for the subsequent classifier models.

#### Radiomics features

We used PyRadiomics [14], an open source package recommended for standardized radiomics analysis workflow [15], to extract 120-deminsional radiomics features of the segmented lung lesions. We extracted the following radiomics features: 19 first-order statistics features, 16 shape-based 3D features, 10 shape-based 2D features, 24 gray-level co-occurrence features, 16 gray-level run length features, 16 gray-level size zone features, five neighboring gray tone difference features, and 14 gray-level dependence features. Details of the feature extraction are presented on the webpage of PyRadiomics [16].

#### Classifier models

**Fig. 1:**
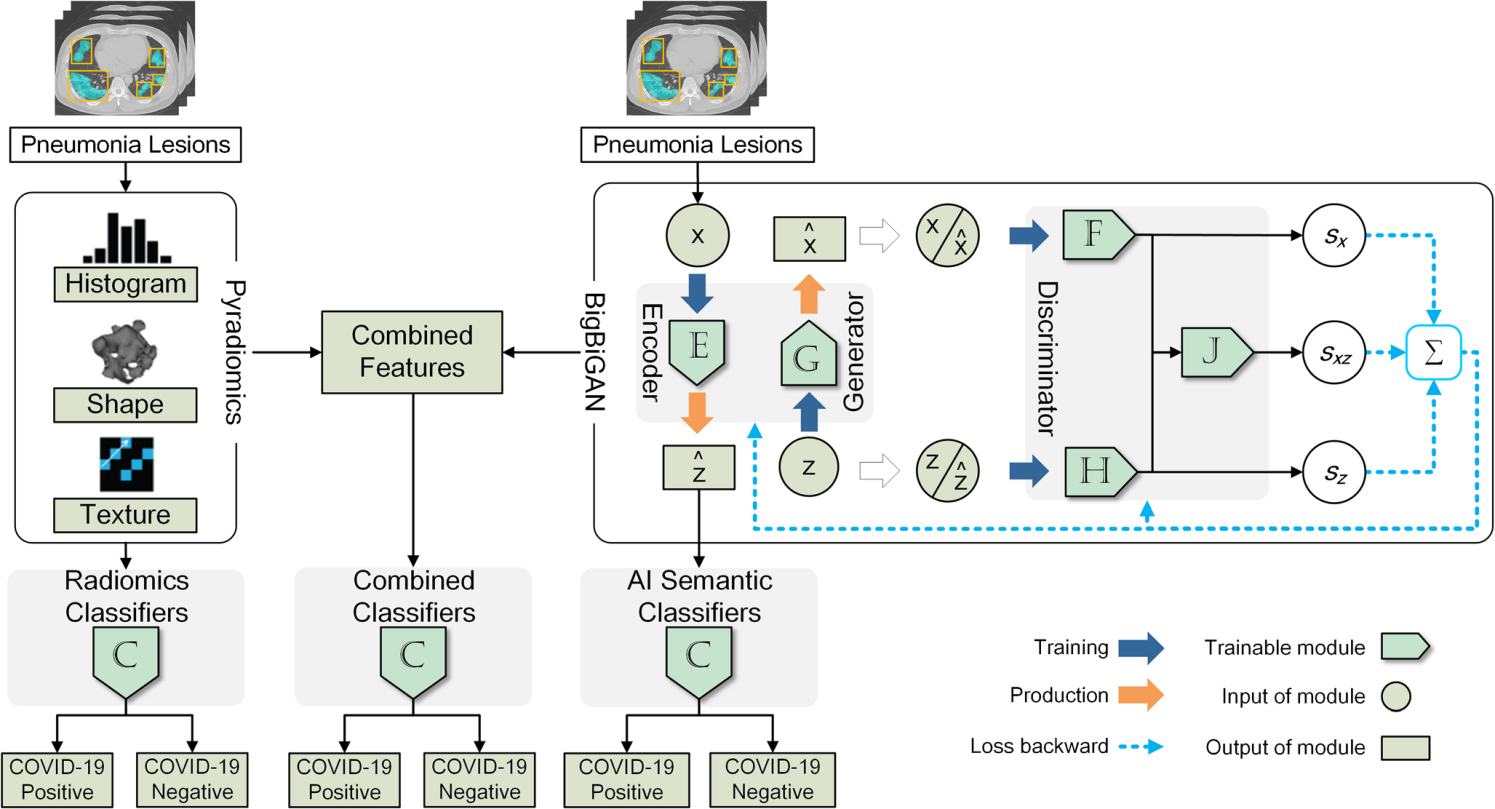
Radiomics and artificial intelligence neural network workflow in this study

To determine performance of DL and radiomics-extracted features, we used two widely used classifiers: a linear classifier typically used in supervised learning, and least absolute shrinkage and selection operator (Lasso) often used in radiomics [17–19]. In addition, we combined both the DL and radiomics features as a single input to determine performance for each of the two models. Model performance was evaluated on hold-out test set, as well as external validation set. The overall study design is illustrated in Fig. 1.

### AI augmentation for clinical diagnosis

Three blinded radiologists from an independent hospital in China reviewed the CT images from the test dataset and external validation dataset and performed the first round of diagnosis for COVID-19 versus non-COVID-19 pneumonia. The radiologists were blinded to clinical diagnosis and RT-PCR test results. After a 2-week wash out period, the reviewers were provided model predictions of *combined deep learning and radiomics features* for a second round of review. Clinical performance with and without model was calculated.

### Statistics analysis

The receiver operating characteristic (ROC) curve and area under curve (AUC), sensitivity, and specificity were used to evaluate the diagnostic accuracy for COVID-19 pneumonia. All statistical computing was performed using R language (version 3.4.3, Vienna, Austria). Based on the image features extracted by PyRadiomics and BigBiGAN, the linear classifier and Lasso were implemented by the “lm()” and “glmnet(),” respectively, for the significant feature selection. Chi-square and ANOVA tests were used to evaluate the differences in demographics. To determine interobserver variability of radiomics features regarding manual segmentation, a new radiologist performed manual segmentation of lung lesions in 10 random patients. Mann-Whitney *U* test was performed on the radiomics features extracted from the two set of manual segmentations. *P* < 0.05 was considered a significant difference.

## Results

### Study cohort

**Fig. 2:**
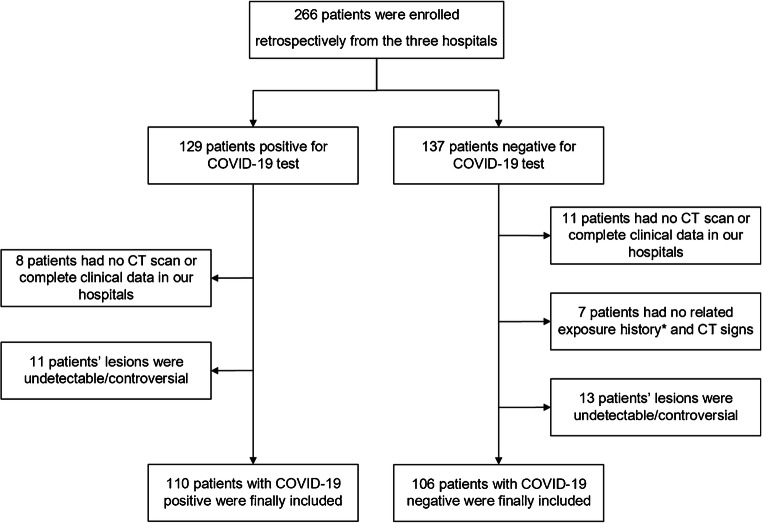
Patient enrollment in our study. Asterisk denotes the exposure history defined in our study (for patients from China): history of travel to Wuhan in the last 14 days, history of contact with confirmed COVID-19 patient(s), and history of being in a dense crowd. The relevant exposure history was selected as an inclusion criterion since these patients were high-risk of COVID-19 infection during this period

A total of 266 patients were initially collected in this study. Ninety-three consecutive COVID-19-positive and 91 COVID-19-negative pneumonia patients from The First Affiliated Hospital of University of Science and Technology of China and The Lu’an Affiliated Hospital of Anhui Medical University in China (January 18–February 29, 2020) met the inclusion criteria. Seventeen patients with COVID-19 pneumonia and 15 patients with COVID-19-negative viral pneumonia from Stanford University Hospital (February 1–May 30, 2020) served as external validation. The flowchart of patient enrollment is shown in Fig. 2.

The mean patient age was 45 years (standard deviation 15.6) with no significant difference between male (*n* = 128) and female (*n* = 88) (*P* > 0.05). The median time interval from symptom onset to CT was 8 days for both COVID-19-positive and -negative patients. The chief complaints of the patients were cough and fever, comprising 95.4%. Detailed demographics are shown in Table 1.

**Table 1:** Demographics of the patients enrolled from the three hospitals in this study

	Hospital 1	Hospital 2	Hospital 3
Patients (total)	144	40	32
No. of COVID-19 positive	73	20	17
No. of COVID-19 negative	71	20	15
Age median (SD)	43 (12.0)	37 (12.1)	59 (15.0)
Sex			
Male	84	28	16
Female	60	12	16
Related exposure history			
History to Wuhan	45	15	*NA*
Contact with infection	21	11	*NA*
Contact with dense crowd	70	30	*NA*
Time interval (median)	5	4	12
Illness classification			
Mild	6	0	*NA*
Common	47	18	*NA*
Severe	20	2	*NA*
Critical illness	0	0	*NA*
Basic disease (yes)	53	5	15
Radiologists’ label slices	11,071	930	3799

*NA*, not applicable

### Chest CT dataset

CT scan details of the study population are shown in Supplementary Material Appendix A. Within the discovery cohort, ten COVID-19-positive and 12 COVID-19-negative patients who did not have visible lung lesions and were excluded from segmentation. Two patients’ lesion segmentations were controversial in radiologists and were excluded. This resulted in a total of 12,001 CT slices (7173 of COVID-19 positive and 4828 of COVID-19 negative) and 24,216 pneumonia lesion segmentations. All slices were randomly divided into training (9, 573), validation (1209), and test (1219 images). The external validation comprised 3799 images (2349 COVID-19 positive and 1450 COVID-19 negative) containing 7883 lesion segmentations of 17 COVID-positive and 15 COVID-negative pneumonia patients. No significant difference was found in the radiomics features extracted from the two sets of manually segmented pneumonia images of the 10 random patients by Mann-Whitney *U* test (*P >* 0.05).

### Image features and model performance

**Fig. 3:**
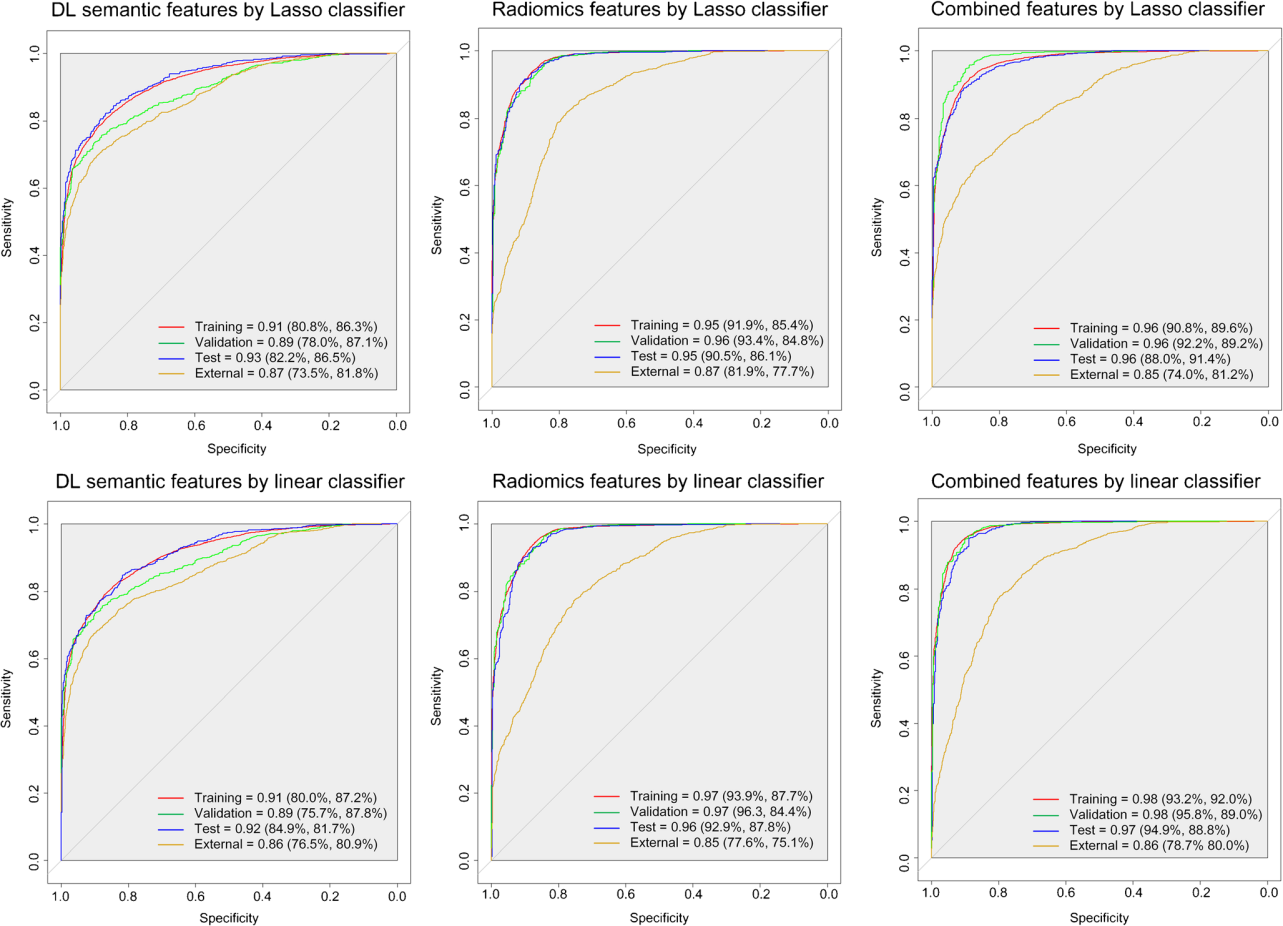
The pneumonia lesions on the CT image were used as the input of the BigBiGAN and PyRadiomics. Receiver operating characteristic curves (ROC) and area under curve (AUC) of the linear classifier and Lasso classifier for the differentiation of COVID-19 from other forms of viral pneumonia with clinical symptoms and CT signs similar to those of COVID-19. The four ROC curves in each chart represent the training (red), validation (green), test (blue), and external validation datasets (yellow), respectively

The AUCs (sensitivity and specificity) of linear and Lasso classifiers using 120-dimensional DL features, 120-dimensional radiomics features, and combined 240-dimensional DL and radiomics features are shown in Fig. 3. Individual DL performances using the whole lung images as inputs are also shown in Supplementary Figure S3. Although the AUCs (sensitivity and specificity) using the whole lung were higher than the pneumonia lesion on the training dataset (linear classifier 0.98 [91.8%, 93.4%] vs. 0.91 [80.0%, 87.2%] and Lasso classifier: 0.97 [93.0%, 92.1%] vs. 0.91 [80.8%, 86.3%]), its performance on the external validation dataset was slightly inferior (linear classifier 0.84 [75.7%, 76.8%] vs. 0.86 [76.5%, 80.9%], and Lasso classifier 0.83 [71.2%, 81.0%] vs. 0.87 [73.5%, 81.8%]).

**Fig. 4:**
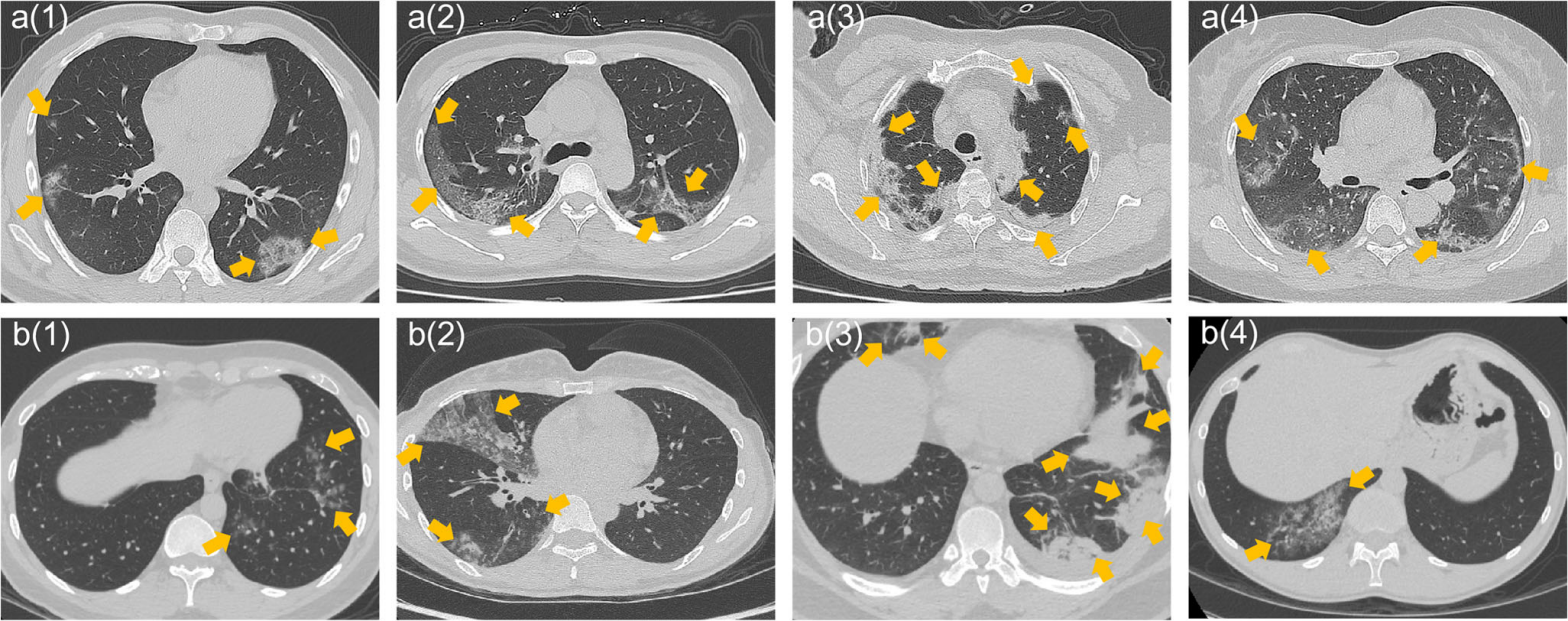
The CT images of COVID-19 positive (**a**) and COVID-19 negative (**b**) with significant different signature values based on the combined feature matrix. Figure a(1) represents a 35-year-old male and CT manifested as bilateral opacities, and linear signature score of 1.32 and Lasso signature score of 0.99; figure a(2) denotes a 43-year-old female and CT manifestation are bilateral ground-glass opacities, vascular thickening, and interlobular septal thickening, with signature scores of 1.23 and 0.99; figure a(3) denotes a 62-year-old male and CT manifestation is bilateral multifocal consolidations. Signature scores are 1.24 and 0.98; figure a(4) represents a 45-year-old female and CT manifested as bilateral peripheral multifocal lesions with signature scores of 1.25 and 0.97; figure b(1) represents a 29-year-old male and CT manifestation is multifocal ground-glass opacities in the left lung. Signature scores are − 0.14 and 0.04; figure b(2) represents a 30-year-old female and CT manifestation is multifocal, mixed ground-glass opacity and consolidation in the right lung. Signature scores are − 0.11 and 0.08; figure b(3) represents a 30-year-old male and CT manifestation is bilateral multifocal consolidation. Signature scores are 0.07 and 0.70; figure b(4) represents a 29-year-old male and CT manifested as mixed densities in the right lung. Signature scores are − 0.17 and 0.03, respectively

Based on the two classifiers, 32 high-dimensional deep learning features were filtered as the significant features (*P* < 0.0001) for the classifying COVID-19 pneumonia, and four radiomics features (*P* < 0.0001), specifically, *mean intensity values* of the lesion and *texture* features (RMS: feature of “original_firstorder_RootMeanSquared; Uniformity: feature of “original_firstorder_Uniformity; NGTDMB: feature of “original_ngtdm_Busyness) were associated with COVID-19 differentiation. The loss curve of the BigBiGAN training is shown in Supplementary Figure S4. Details of the significant features are shown in Supplementary Material Appendix B and C. When using the combined 240-dimensional DL and radiomics features, the following features were selected by both classifiers (*P* < 0.0001): five radiomics features (diagnostics_Image-original_Mean, original_shape_Maximum2DDiameterSlice, original_firstorder_Skewness, original_firstorder_Uniformity, and original_ngtdm_Busyness) and 6 deep learning features (18th, 24th, 35th, 50th, 65th, and 79th feature). The distribution of the values of these features in COVID-19-positive and -negative images is shown in Supplementary Material Appendix D. The signatures constructed by the linear classifier and Lasso classifier based on the combined features are shown in Supplementary Figure S5; CT images of COVID-19 and non-COVID-19 with significant image signature values are shown in Fig. 4.

### Clinical use

**Fig. 5:**
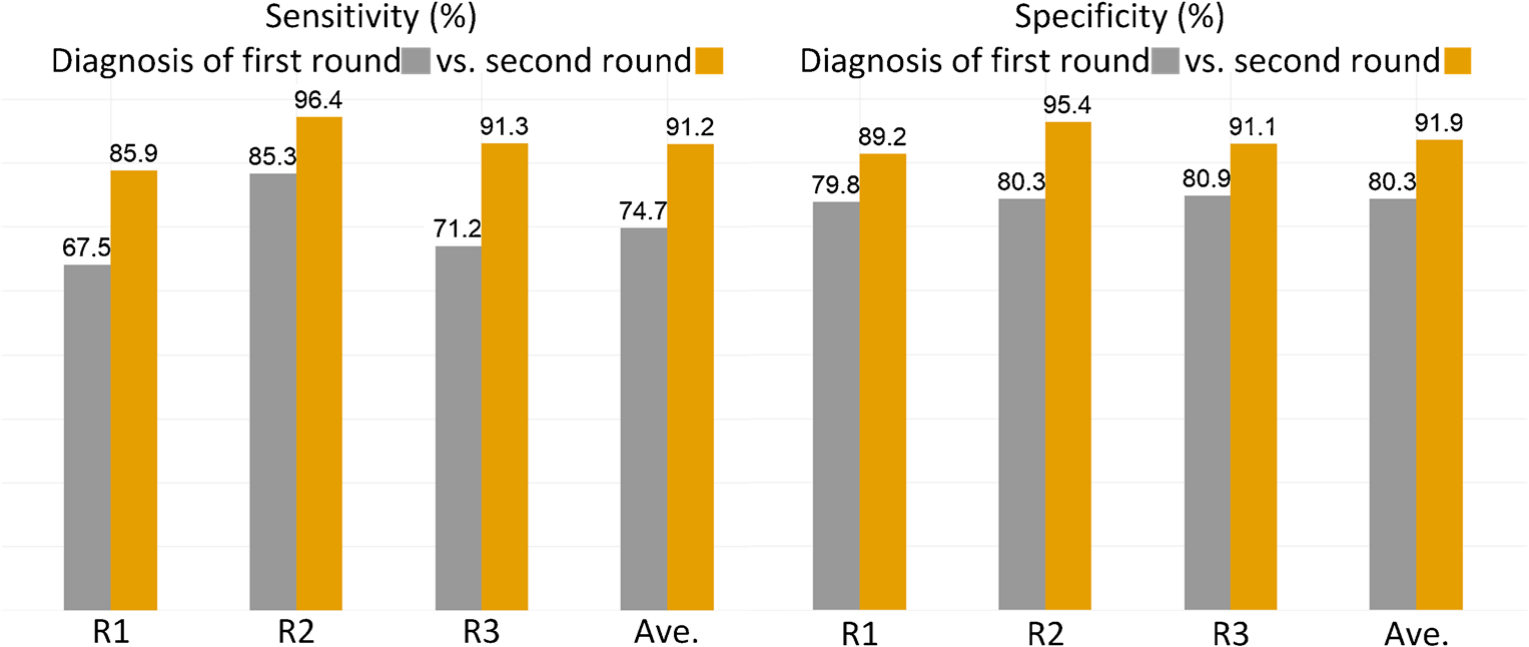
Sensitivity and specificity of the radiologists’ diagnosis on the test datasets without (first round of diagnosis) and with (second round of diagnosis) the assistance of our AI semantic features plus radiomics features

Diagnostic sensitivity and specificity of 3 radiologists on two test datasets were 74.7% and 80.3%, respectively. With the prediction outputs from *combined deep learning and radiomics features*, their performance increased with sensitivity and specificity of 91.2% and 91.9%, respectively, as shown in Fig. 5.

## Discussion

In this study, we uncover some of the deep learning and radiomics features that contribute to differentiation of COVID-19 from non-COVID-19 viral pneumonia. Features extracted from both deep learning and radiomics showed similar performance with linear and Lasso classifiers, with sensitivity > 73% and specificity > 75% on the external cohort. DL features extracted from pneumonia lesion performed superior to the whole lung on the external validation dataset. Prediction outputs generated from our combined deep learning-radiomics model further augmented human expert performance.

To our knowledge, this is the first study to compare performance of DL versus radiomics models for differentiation of COVID-19 pneumonia. Various studies have described performance of DL models for COVID-19 pneumonia [20, 21]. However, specific image features relevant to COVID-19 classification remain opaque. While it is well-known that with large datasets, DL models perform superior to hand-crafted feature extraction [22, 23], large data are not always possible in medicine and may be limited by disease prevalence, obstacles to data procurement, and other clinical factors. For smaller data, studies have suggested feature engineering may be a more suitable machine learning strategy with notable advantages of radiomics for medical imaging analysis [15, 24, 25]. At present, studies that directly compare radiomics and deep learning clinical model performance are relatively unexplored [26]. In this study, we address these questions and further aim to enhance interpretability of such machine learning models.

Recent studies have shown image features learned from a BigBiGAN framework can achieve state-of-the-art performance for image classification [11, 12, 27]. Unlike traditional generative adversarial network typically used for image synthesis, de-noising, or generation of high-quality images, BigBiGAN has shown robust performance for learning high-dimensional semantic features. We identified 32 deep learning features that differed significantly between COVID-19-positive and -negative lesion images (*P* < 0.0001). Using PyRadiomics analysis, four radiomics features were selected by the two classifiers (*P* < 0.0001) to differentiate COVID-19 from other types of viral pneumonia. When we combined both approaches, 6 deep learning features and 5 radiomics features were selected (*P* < 0.0001) by the two classifiers. These results might suggest more distinguishing features were learned on neural network. Although ROC might suggest slightly more robust performance for radiomics models, sensitivity and specificity did not differ among deep learning, radiomics, and combined features.

Among the four significant radiomics features, we found *mean intensity* of COVID-19 (− 665.9) lesions to be higher than non-COVID-19 lesions (− 887.0) which might reflect more diffuse opacities or greater degree of fluid or debris affecting the airspaces. Based on NGTDMB, which measures the rate of change in intensity between pixels and its neighborhoods, we also found less intense change between adjacent pixels for COVID-19 (0.39) compared to non-COVID-19 (0.72), which might also indicate that within an affected lung region, there is more diffuse airspace process, *sparing fewer* of the neighboring alveoli in COVID-19 compared to other types of viral pneumonia. This might also explain why RMS, which measures the magnitude of the image values by calculating contributions of each gray value (absolute), was higher for non-COVID-19 (660.8 vs. 563.1), where greater number of spared alveoli, i.e., air-filled, rather than alveoli fluid-filled by disease, could contribute to a wider magnitude of absolute gray values in the pixels. Uniformity was lower for COVID-19 (0.03) compared to non-COVID-19 (0.06) lesions, with a larger range in irregular texture for COVID-19 and suggested a more heterogeneous lung texture possibly due to diversity in airspace disease phenotypes (consolidation, ground-glass opacities, etc.) that combine varying degrees of edema and vascular and interlobular septal thickening, sometimes described as “crazy paving” on visual inspection [28].

Although it is difficult to directly map image phenotype from DL features alone, signatures from combined DL and radiomics features provide some clues to image-based discrimination for COVID-19 versus non-COVID-19 lung disease. COVID-19 patients showed higher signature scores for *irregular intensity changes*, *heterogeneous intensities*, and *wider range in textures* within the lung lesions compared with non-COVID-19 patients. For example, despite a large area of mixed opacity that might raise suspicion for COVID-19 on visual inspection (Fig. 4b(2)), feature extraction revealed *intensity changes that were relatively regular* within the lesion, a pattern that was associated with non-COVID-19. In another example, although multiple, nodular opacities in peripheral consolidative pattern on CT might raise suspicion for COVID-19 (Fig. 4b(3)), *strong uniformity of CT intensity values* within the lesions suggested a non-COVID-19 process. This was supported by the linear signature score (0.07) that was consistent with non-COVID-19.

When predictions from a combined deep learning-radiomics model were available, we observed improved radiologist diagnostic performance with increase in both sensitivity and specificity by 16.5% and 11.6%, respectively, suggesting a potential role for machine learning for augmenting clinician decision support.

There are several limitations to this study. While we used the high-dimensional, semantic features from the encoder module of the BigBiGAN framework, we did not examine other features that can be produced in the framework or by a different architecture. Since there is no specific definition of these deep learning semantic features, in the future, we will explore image encoding processes used to generate each of the deep learning semantic features to further enhance interpretability of these features. While deep learning and radiomics approaches performed comparably with a training cohort of around 180 patients with 9500 images, larger dataset could show more robust performance for deep learning. Finally, while we used features when the loss was minimum in the last training epoch, “deep learning features” might vary over training parameters or change with other experimental settings.

In conclusion, we uncover specific deep learning and radiomics features relevant to COVID-19 pneumonia to assist interpretability of machine learning algorithms and contribute to understanding of COVID-19 pneumonia imaging phenotypes. Furthermore, we compare performance of deep learning and radiomics models for COVID-19 pneumonia diagnosis using chest CT and show potential for augmenting radiologist diagnostic performance with the aid of machine learning predictions.

## Electronic supplementary material

ESM 1 (DOCX 15.7 mb)

## Data Availability

The data link: http://dx.doi.org/10.17632/yn7vpd7bxk.1

## References

[1] WHO. Coronavirus disease (COVID-2019) situation reports. Coronavirus disease (COVID-2019) situation reports. World Health Organization; 2020. https://www.who.int/emergencies/diseases/novel-coronavirus-2019.

[2] Li Z, Yi Y, Luo X, Xiong N, Liu Y, Li S, et al. Development and clinical application of a rapid IgM-IgG combined antibody test for SARS-CoV-2 infection diagnosis. J Med Virol. 2020. 10.1002/jmv.25727. 10.1002/jmv.25727 PMC7228300 32104917

[3] Rivett L, Sridhar S, Sparkes D, Routledge M, Jones NK, Forrest S, et al. Screening of healthcare workers for SARS-CoV-2 highlights the role of asymptomatic carriage in COVID-19 transmission. Elife. 2020;9. 10.7554/eLife.58728. 10.7554/eLife.58728 PMC7314537 32392129

[4] Fang Y, Zhang H, Xie J, Lin M, Ying L, Pang P, et al. Sensitivity of chest CT for COVID-19: comparison to RT-PCR. Radiology. 2020;200432. 10.1148/radiol.2020200432 PMC7233365 32073353

[5] Ai T, Yang Z, Hou H, Zhan C, Chen C, Lv W, et al. Correlation of chest CT and RT-PCR testing in coronavirus disease 2019 (COVID-19) in China: a report of 1014 cases. Radiology. 2020;200642. 10.1148/radiol.2020200642 PMC7233399 32101510

[6] Bernheim A, Mei X, Huang M, Yang Y, Fayad ZA, Zhang N, et al. Chest CT findings in coronavirus disease-19 (COVID-19): relationship to duration of infection. Radiology. 2020;200463. 10.1148/radiol.2020200463 PMC7233369 32077789

[7] Bai HX, Hsieh B, Xiong Z, Halsey K, Choi JW, Tran TML, et al. Performance of radiologists in differentiating COVID-19 from viral pneumonia on chest CT. Radiology. 2020;200823. 10.1148/radiol.2020200823. 10.1148/radiol.2020200823 PMC7233414 32155105

[8] Wang S, Zha Y, Li W, Wu Q, Li X, Niu M, et al. A fully automatic deep learning system for COVID-19 diagnostic and prognostic analysis. Eur Respir J. 2020. 10.1183/13993003.00775-2020. 10.1183/13993003.00775-2020 PMC7243395 32444412

[9] Li L, Qin L, Xu Z, Yin Y, Wang X, Kong B, et al. Artificial intelligence distinguishes COVID-19 from community acquired pneumonia on chest CT. Radiology. 2020;200905. 10.1148/radiol.2020200905. 10.1148/radiol.2020200905 PMC7233473 32191588

[10] Zhang L, Wang DC, Huang Q, Wang X (2020). Significance of clinical phenomes of patients with COVID-19 infection: a learning from 3795 patients in 80 reports. Clin Transl Med.

[11] Donahue J, Simonyan K. Large scale adversarial representation learning. Adv Neural Inf Proces Syst; 2019. p. 10541–51.

[12] Song J, Wang H, Liu Y, Wu W, Dai G, Wu Z, et al. End-to-end automatic differentiation of the coronavirus disease 2019 (COVID-19) from viral pneumonia based on chest CT. Eur J Nucl Med Mol Imaging 2020. doi:10.1007/s00259-020-04929-1. 10.1007/s00259-020-04929-1 PMC7306401 32567006

[13] Google Colaboratory. GOOGLE; 2017. https://colab.research.google.com/.

[14] van Griethuysen JJM, Fedorov A, Parmar C, Hosny A, Aucoin N, Narayan V (2017). Computational radiomics system to decode the radiographic phenotype. Cancer Res.

[15] Lambin P, Leijenaar RT, Deist TM, Peerlings J, De Jong EE, Van Timmeren J (2017). Radiomics: the bridge between medical imaging and personalized medicine. Nat Rev Clin Oncol.

[16] Joost van Griethuysen AF, Aucoin N, Fillion-Robin J-C, Hosny A, Pieper S, Aerts H. PyRadiomics: radiomic features. 2020.

[17] Jiang Y, Chen C, Xie J, Wang W, Zha X, Lv W (2018). Radiomics signature of computed tomography imaging for prediction of survival and chemotherapeutic benefits in gastric cancer. EBioMedicine..

[18] Jiang Y, Wang W, Chen C, Zhang X, Zha X, Lv W (2019). Radiomics signature on computed tomography imaging: association with lymph node metastasis in patients with gastric cancer. Front Oncol.

[19] Wei W, Liu Z, Rong Y, Zhou B, Bai Y, Wei W (2019). A computed tomography-based radiomic prognostic marker of advanced high-grade serous ovarian cancer recurrence: a multicenter study. Front Oncol.

[20] Mei X, Lee HC, Diao KY, Huang M, Lin B, Liu C, et al. Artificial intelligence-enabled rapid diagnosis of patients with COVID-19. Nat Med. 2020. 10.1038/s41591-020-0931-3. 10.1038/s41591-020-0931-3 PMC7446729 32427924

[21] Belfiore MP, Urraro F, Grassi R, Giacobbe G, Patelli G, Cappabianca S (2020). Artificial intelligence to codify lung CT in Covid-19 patients. Radiol Med.

[22] Korotcov A, Tkachenko V, Russo DP, Ekins S (2017). Comparison of deep learning with multiple machine learning methods and metrics using diverse drug discovery data sets. Mol Pharm.

[23] Moen E, Bannon D, Kudo T, Graf W, Covert M, Van Valen D (2019). Deep learning for cellular image analysis. Nat Methods.

[24] Song J, Shi J, Dong D, Fang M, Zhong W, Wang K (2018). A new approach to predict progression-free survival in stage IV EGFR-mutant NSCLC patients with EGFR-TKI therapy. Clinical cancer research : an official journal of the American Association for Cancer Research.

[25] Colen RR, Fujii T, Bilen MA, Kotrotsou A, Abrol S, Hess KR (2018). Radiomics to predict immunotherapy-induced pneumonitis: proof of concept. Investig New Drugs.

[26] Choi JY (2018). Radiomics and deep learning in clinical imaging: what should we do?. Nucl Med Mol Imaging.

[27] Mozafari M, Reddy L, VanRullen R. Reconstructing natural scenes from fMRI patterns using BigBiGAN. arXiv preprint arXiv:200111761. 2020.

[28] Han R, Huang L, Jiang H, Dong J, Peng H, Zhang D. Early clinical and CT manifestations of coronavirus disease 2019 (COVID-19) Pneumonia. AJR Am J Roentgenol. 2020:1–6. 10.2214/AJR.20.22961. 10.2214/AJR.20.22961 32181672

