# Supplementary material for: Decoding COVID-19 pneumonia: comparison of deep learning and radiomics CT image signatures
Source: Eur J Nucl Med Mol Imaging. 2020 Oct 23;48(5):1478–86. doi: 10.1007/s00259-020-05075-4 (PMC7581467; doi:10.1007/s00259-020-05075-4)
Supplement: Supplementary file 1 — (DOCX 15.7 mb) [file 259_2020_5075_MOESM1_ESM.docx]

**Supplementary Materials**

**Decoding COVID-19 Pneumonia: Comparison of Deep Learning and Radiomics CT Image Signatures**

**Figure S1.** Procedure of the manual segmentation of pneumonia lesions by ITK-Snap.

**Figure S2.** Examples of the pneumonia lesion segmentations used for training and feature extraction.

**Figure S3.** The AUCs (sensitivity and specificity) of linear and Lasso classifiers of individual deep learning performances using the whole lung CT image.

**Figure S4.** The loss curve of the training of the BigBiGAN.

**Figure S5.** Distribution of the values of the signature constructed by the Lasso classifier and linear classifier based on the deep learning image features plus radiomic features which extracted from the COVID-19 and non-COVID-19 CT images.

**Appendix A.** CT scan details of the study population.

**Appendix B.** Four radiomic features filtered as the significant features (*P* < 0.0001) for the classifying COVID-19 pneumonia.

**Appendix C.** 32 high-dimensional, semantic features from BigBiGAN deep learning framework (*P* < 0.0001) for the classifying COVID-19 pneumonia.

**Appendix D.** The distribution of the values of the features selected by combined model in COVID-19 positive and negative images.

**
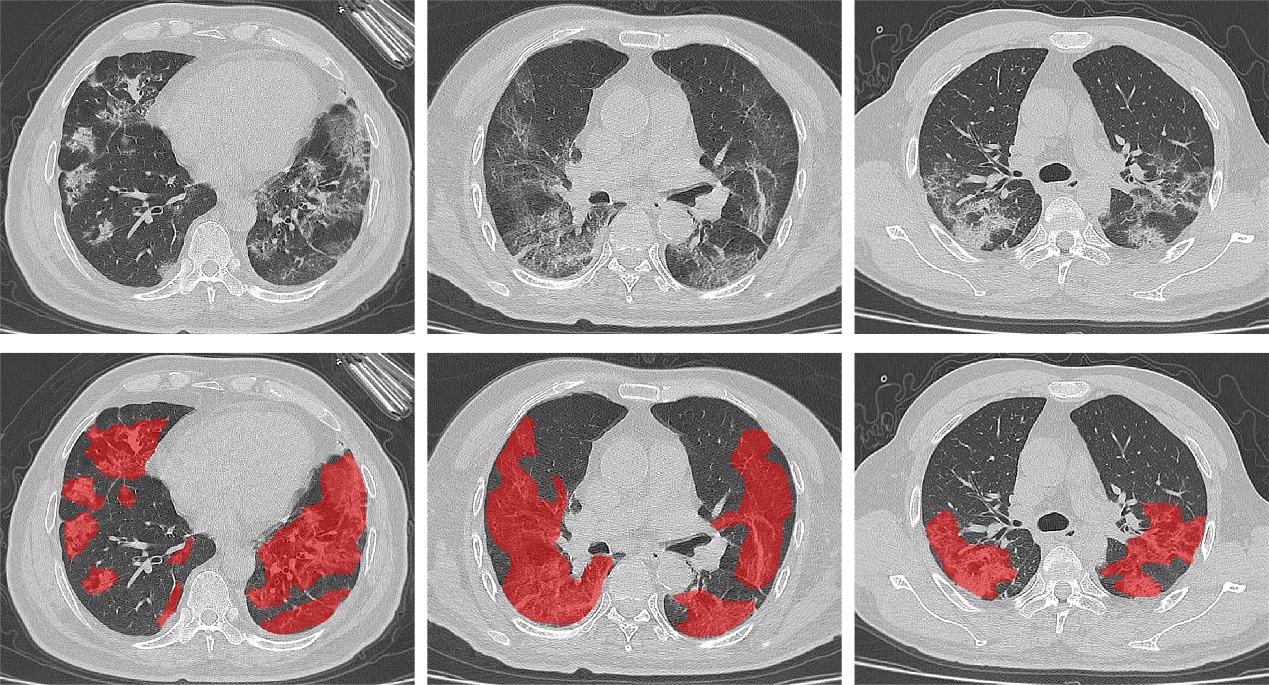
**

**Figure S1.** Procedure of the manual segmentation of pneumonia lesions by ITK-Snap. The first row are the original CT images, and the second row are the corresponding masks of the pneumonia lesions by radiologists. First, the radiologist outlined the boundary points of each lesion on the original CT images, and the mask (in red) was then automatically generated by ITK-Snap after the outline of a lesion was completely delineated. The procedure was repeated slice-by-slice until all the CT slices with pneumonia lesions were processed.

**
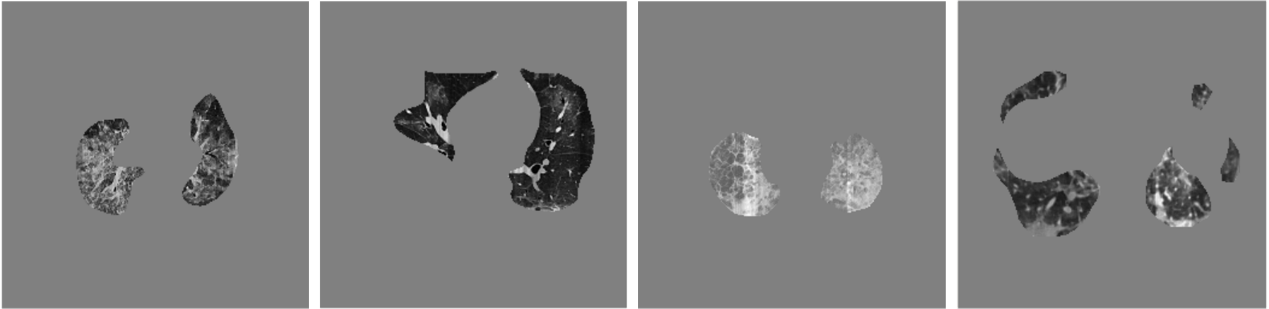
**

**Supplementary Figure S2.** Examples of the pneumonia lesion segmentations used for training and feature extraction.

**
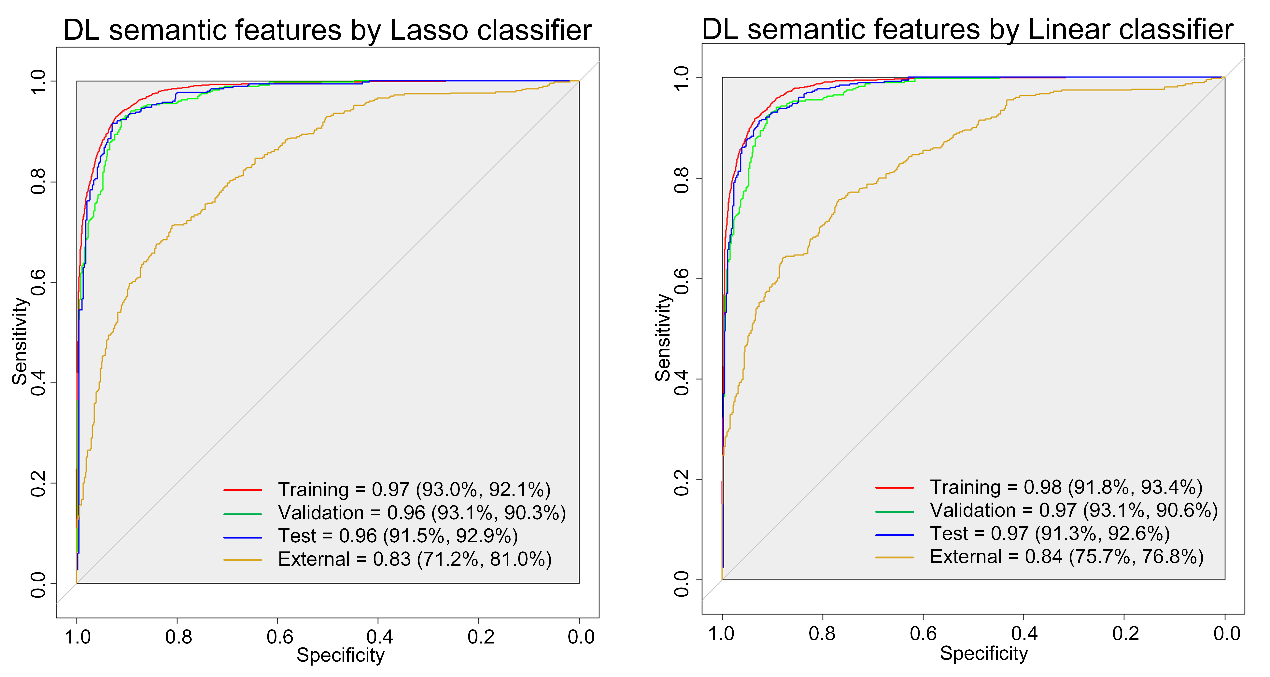
**

**Supplementary Figure S3.** The whole CT image was used as the input of the BigBiGAN. Receiver operating characteristic curves (ROC) and area under curve (AUC) of the linear classifier and Lasso classifier for the differentiation of COVID-19 from other forms of viral pneumonia with clinical symptoms and CT signs similar to those of COVID-19.

**
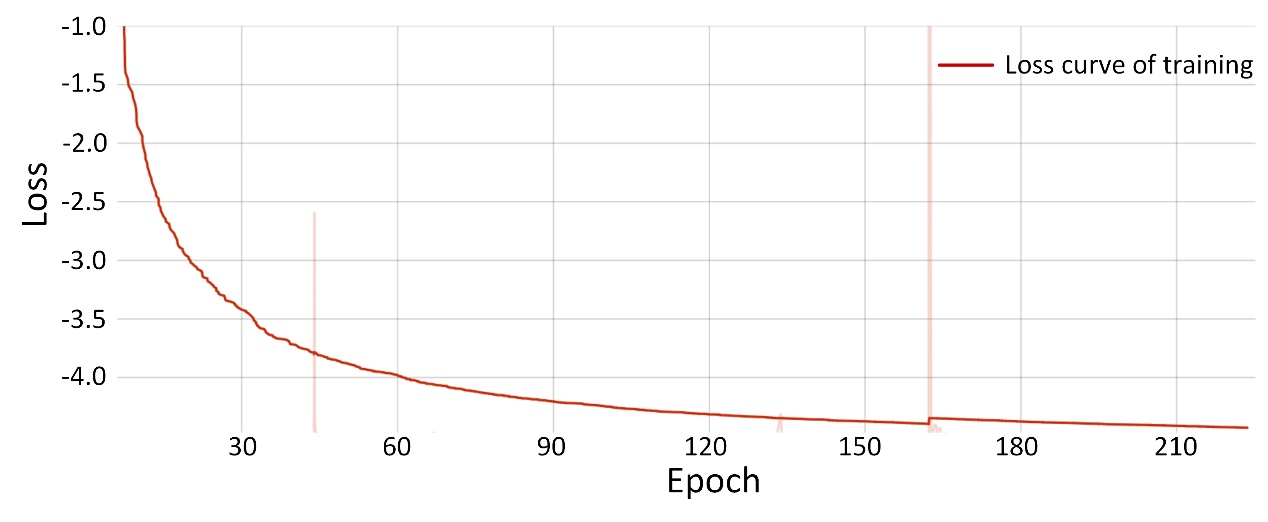
**

**Supplementary Figure S4.** The loss curve of the training of the BigBiGAN. The curve has been smoothed for better visualization. There are some translucent outliers because rental time limitation of the online Google Colab.

**
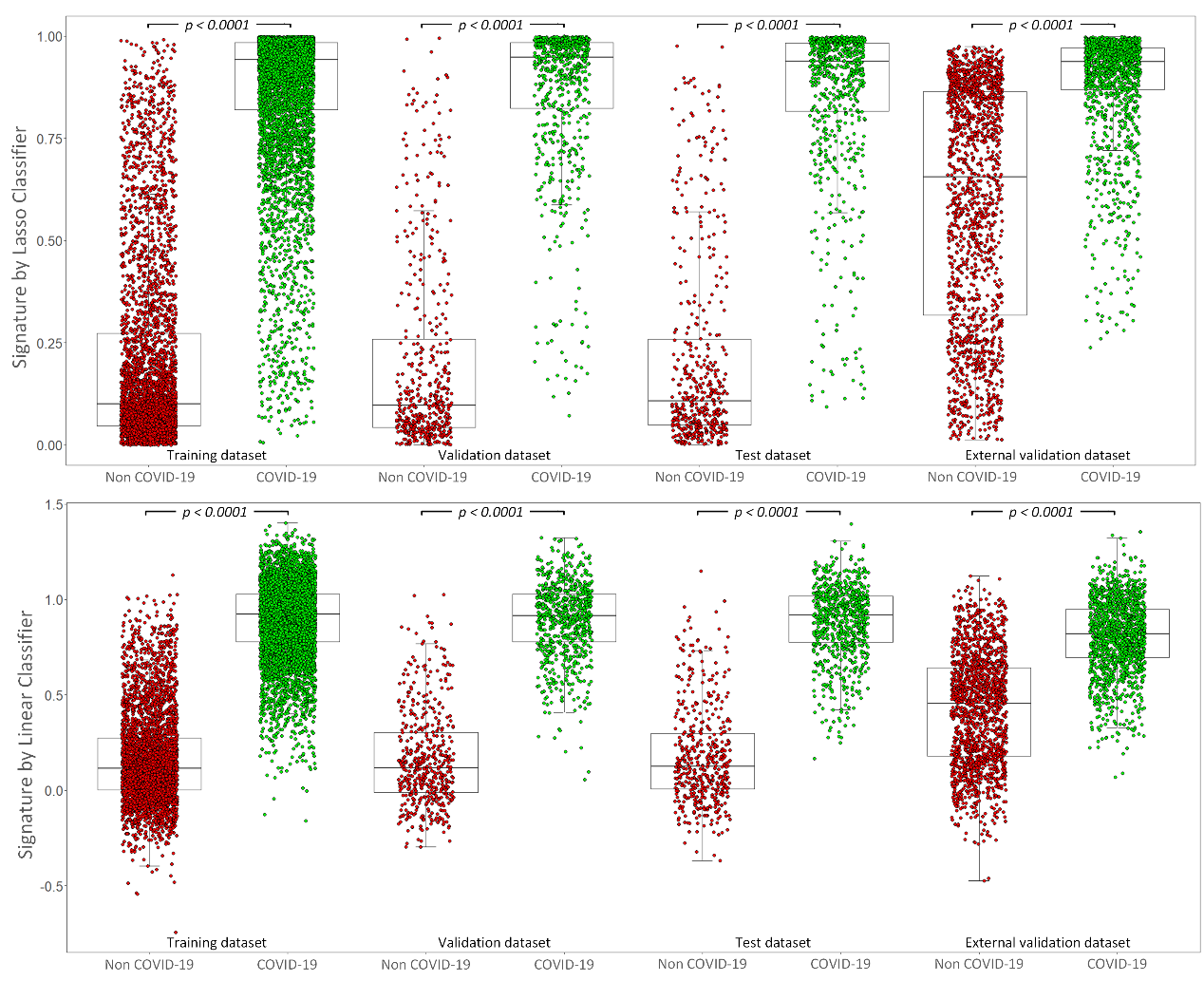
**

**Supplementary Figure S5.** Distribution of the values of the signature constructed by the Lasso classifier and linear classifier based on the deep learning semantic features plus radiomic features which extracted from the COVID-19 and non-COVID-19 CT images.

**Appendix A**

In the study population, 68 patients from The First Affiliated Hospital of University of Science and Technology of China were imaged with a CT slice of 1-mm thickness on a GE Revolution 256 scanner (GE Medical Systems, Waukesha, USA), and 76 patients were imaged with a CT slice of 5-mm thickness on a NeuViz 128 scanner (Neusoft, Shenyang, China). A total of 40 patients from The Lu ’an affiliated hospital of Anhui Medical University were imaged with a CT slice of 5-mm thickness on a NeuViz 64 scanner (Neusoft, Shenyang, China). CT scans of the other 32 patients from Stanford University Hospital as the external validation dataset were obtained at 1-3 mm slice thickness with or without contrast (Lightspeed VCT and Revolution, GE Healthcare, Milwaukee, WI; Aquilion, Toshiba Medical Systems, Otawara, Japan; SOMATOM, Siemens Healthineers, Erlangen, Germany).

**Appendix B**

Significant features selected by both the linear classifier and Lasso classifier based on the 120-dimensional radiomic features. The data of “COVID-19 vs. Non-COVID-19” represents the mean values of images with COVID-19 positive versus COVID-19 negative. All of the four features are significant in the training, validation, test, and external validation dataset (*P* < 0.0001). T test is used to analyze the statistical difference between the two.

|  | Training dataset | | Validation dataset | | Test dataset | | External validation dataset | |
| --- | --- | --- | --- | --- | --- | --- | --- | --- |
|  | COVID-19 vs.  Non-COVID-19 | *P* (t test) | COVID-19 vs.  Non-COVID-19 | *P* (t test) | COVID-19 vs.  Non-COVID-19 | *P* (t test) | COVID-19 vs.  Non-COVID-19 | *P* (t test) |
| Mean | -707.47 vs. -999.35 | <0.0001 | -709.84 vs. -998.32 | <0.0001 | -715.97 vs. -995.68 | <0.0001 | -530.32 vs. -554.74 | <0.0001 |
| RMS | 585.44 vs. 664.73 | <0.0001 | 586.69 vs. 666.21 | <0.0001 | 583.73 vs. 661.52 | <0.0001 | 496.43 vs. 651.03 | <0.0001 |
| Uniformity | 0.03 vs. 0.06 | <0.0001 | 0.03 vs. 0.06 | <0.0001 | 0.03 vs. 0.06 | <0.0001 | 0.03 vs. 0.06 | <0.0001 |
| NGTDMB | 0.36 vs. 0.56 | <0.0001 | 0.35 vs. 0.54 | <0.0001 | 0.40 vs. 0.58 | <0.0001 | 0.47 vs. 1.21 | <0.0001 |

Note: Mean: feature of “diagnostics_Image-original_Mean” in Pyradiomics, RMS: feature of “original_firstorder_RootMeanSquared”, Uniformity: feature of “original_firstorder_Uniformity”, NGTDMB: feature of “original_ngtdm_Busyness”.

**Appendix C**

32 high-dimensional, semantic features from BigBiGAN deep learning framework (P < 0.0001) for the classifying COVID-19 pneumonia. T test is used to analyze the statistical difference. The data of “COVID-19 vs. Non-COVID-19” represents the mean values of images with COVID-19 positive versus COVID-19 negative. ***** denotes the significant difference of the features in all the four datasets are *P* < 0.0001.

|  | Training dataset | | Validation dataset | | Test dataset | | External validation dataset | |
| --- | --- | --- | --- | --- | --- | --- | --- | --- |
|  | COVID-19 vs.  Non-COVID-19 | *P* (t test) | COVID-19 vs.  Non-COVID-19 | *P* (t test) | COVID-19 vs.  Non-COVID-19 | *P* (t test) | COVID-19 vs.  Non-COVID-19 | *P* (t test) |
| ***Feature2** | -0.03 vs. 0.11 | <0.0001 | -0.04 vs. 0.11 | <0.0001 | -0.03 vs. 0.12 | <0.0001 | -0.13 vs. -0.05 | <0.0001 |
| Feature5 | -0.10 vs. -0.62 | <0.0001 | -0.04 vs. -0.67 | <0.0001 | -0.09 vs. -0.64 | <0.0001 | -0.09 vs. -0.11 | 0.4722 |
| ***Feature8** | -0.42 vs. -0.91 | <0.0001 | -0.44 vs. -0.91 | <0.0001 | -0.45 vs. -0.94 | <0.0001 | -0.06 vs. -0.46 | <0.0001 |
| Feature13 | 1.12 vs. 1.15 | <0.0001 | 1.10 vs. 1.16 | <0.0001 | 1.12 vs. 1.15 | 0.0112 | 1.05 vs. 1.08 | 0.0119 |
| ***Feature16** | -0.46 vs. -0.68 | <0.0001 | -0.48 vs. -0.65 | <0.0001 | -0.45 vs. -0.68 | <0.0001 | -0.28 vs. -0.46 | <0.0001 |
| ***Feature17** | 0.16 vs. 0.03 | <0.0001 | 0.16 vs. 0.02 | <0.0001 | 0.14 vs. 0.03 | <0.0001 | 0.17 vs. -0.02 | <0.0001 |
| ***Feature18** | 0.61 vs. 0.48 | <0.0001 | 0.61 vs. 0.48 | <0.0001 | 0.61 vs. 0.49 | <0.0001 | 0.62 vs. 0.37 | <0.0001 |
| Feature19 | 0.29 vs. 0.53 | <0.0001 | 0.29 vs. 0.53 | <0.0001 | 0.28 vs. 0.53 | <0.0001 | 0.23 vs. 0.22 | 0.3120 |
| Feature20 | 0.31 vs. 0.35 | <0.0001 | 0.31 vs. 0.35 | 0.0586 | 0.31 vs. 0.37 | 0.0125 | 0.28 vs. 0.20 | <0.0001 |
| Feature23 | 0.66 vs. 0.91 | <0.0001 | 0.68 vs. 0.92 | <0.0001 | 0.66 vs. 0.92 | <0.0001 | 0.39 vs. 0.46 | 0.0122 |
| ***Feature24** | 0.61 vs. 1.10 | <0.0001 | 0.67 vs. 1.08 | <0.0001 | 0.63 vs. 1.11 | <0.0001 | 0.58 vs. 0.94 | <0.0001 |
| ***Feature33** | -0.35 vs. 0.02 | <0.0001 | -0.35 vs. 0.04 | <0.0001 | -0.37 vs. 0.04 | <0.0001 | -0.41 vs. -0.18 | <0.0001 |
| Feature35 | -0.34 vs. -0.39 | <0.0001 | -0.35 vs. -0.4 | 0.2518 | -0.34 vs. -0.41 | 0.0671 | -0.47 vs. -0.62 | <0.0001 |
| Feature36 | -0.61 vs. -0.52 | <0.0001 | -0.57 vs. -0.56 | 0.9909 | -0.61 vs. -0.50 | 0.0016 | -0.54 vs. -0.46 | 0.0001 |
| Feature38 | -0.27 vs. -0.03 | <0.0001 | -0.33 vs. -0.01 | <0.0001 | -0.26 vs. 0.01 | <0.0001 | -0.34 vs. -0.32 | 0.4590 |
| Feature45 | -0.75 vs. -0.94 | <0.0001 | -0.75 vs. -0.96 | <0.0001 | -0.76 vs. -0.95 | <0.0001 | -0.74 vs. -0.76 | 0.1854 |
| ***Feature50** | -1.37 vs. -1.89 | <0.0001 | -1.36 vs. -1.91 | <0.0001 | -1.38 vs. -1.91 | <0.0001 | -1.27 vs. -1.72 | <0.0001 |
| Feature51 | -0.16 vs. -0.21 | <0.0001 | -0.15 vs. -0.21 | 0.0026 | -0.18 vs. -0.21 | 0.1083 | -0.31 vs. -0.03 | <0.0001 |
| Feature53 | 0.22 vs. 0.26 | <0.0001 | 0.24 vs. 0.20 | 0.2005 | 0.22 vs. 0.28 | 0.0018 | 0.22 vs. 0.32 | <0.0001 |
| Feature54 | 1.00 vs. 0.96 | <0.0001 | 1.07 vs. 0.93 | <0.0001 | 1.02 vs. 0.98 | 0.2143 | 0.91 vs. 0.91 | 0.9342 |
| ***Feature55** | -1.12 vs. -0.96 | <0.0001 | -1.15 vs. -0.96 | <0.0001 | -1.11 vs. -0.95 | <0.0001 | -1.21 vs. -1.09 | <0.0001 |
| Feature56 | -0.07 vs. -0.18 | <0.0001 | -1.15 vs. -0.96 | <0.0001 | -0.10 vs. -0.18 | 0.0007 | -0.07 vs. -0.18 | <0.0001 |
| Feature58 | 0.25 vs. 0.18 | <0.0001 | 0.25 vs. 0.16 | 0.0005 | 0.27 vs. 0.20 | 0.0025 | 0.23 vs. 0.13 | <0.0001 |
| ***Feature63** | -1.66 vs. -1.44 | <0.0001 | -1.69 vs. -1.44 | <0.0001 | -1.65 vs. -1.43 | <0.0001 | -1.85 vs. -1.64 | <0.0001 |
| ***Feature65** | 0.07 vs. 0.31 | <0.0001 | 0.05 vs. 0.29 | <0.0001 | 0.08 vs. 0.34 | <0.0001 | -0.05 vs. -0.17 | <0.0001 |
| Feature66 | 0.26 vs. 0.36 | <0.0001 | 0.27 vs. 0.38 | 0.0011 | 0.29 vs. 0.32 | 0.4084 | 0.17 vs. 0.20 | 0.2378 |
| ***Feature67** | 0.22 vs. -0.08 | <0.0001 | 0.22 vs. -0.01 | <0.0001 | 0.23 vs. -0.10 | <0.0001 | 0.51 vs. 0.04 | <0.0001 |
| ***Feature74** | -1.32 vs. -1.14 | <0.0001 | -1.32 vs. -1.16 | <0.0001 | -1.32 vs. -1.14 | <0.0001 | -1.36 vs. -1.19 | <0.0001 |
| ***Feature79** | 0.20 vs. 0.50 | <0.0001 | 0.22 vs. 0.50 | <0.0001 | 0.20 vs. 0.52 | <0.0001 | 0.09 vs. 0.43 | <0.0001 |
| Feature80 | -1.40 vs. -1.51 | <0.0001 | -1.43 vs. -1.51 | 0.0095 | -1.41 vs. -1.53 | 0.0002 | -1.31 vs. -1.01 | <0.0001 |
| Feature88 | -0.21 vs. -0.01 | <0.0001 | -0.23 vs. -0.02 | <0.0001 | -0.22 vs. 0.00 | <0.0001 | -0.36 vs. -0.35 | 0.4745 |
| Feature89 | 0.38 vs. 0.51 | <0.0001 | 0.39 vs. 0.45 | 0.3080 | 0.41 vs. 0.52 | 0.0693 | 0.36 vs. 0.42 | 0.0856 |

**Appendix D**

Distribution of value of each significant feature in images with COVID-19 positive versus COVID-19 negative which selected by both the linear classifier and Lasso classifier based on the deep learning semantic features plus radiomic features (240-dimensional features in total).


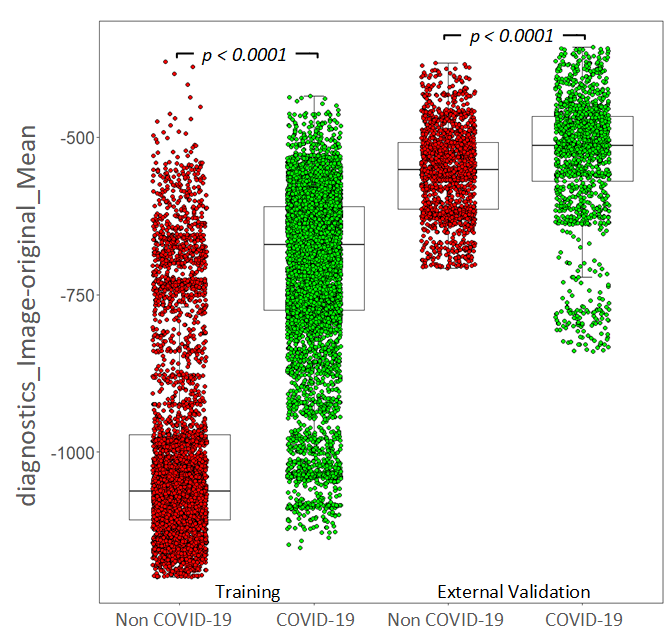


**Appendix D eFigure 1.** Distribution of values of the “diagnostics_Image-original_Mean” feature between the COVID-19 images and non-COVID-19 images in the training and external validation datasets.


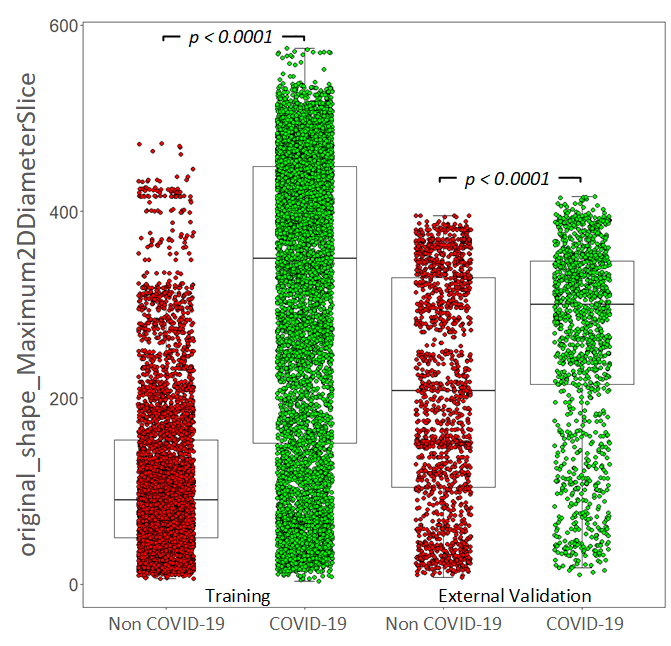


**Appendix D eFigure 2.** Distribution of values of the “original_shape_Maximum2DDiameterSlice” feature between the COVID-19 images and non-COVID-19 images in the training and external validation datasets.


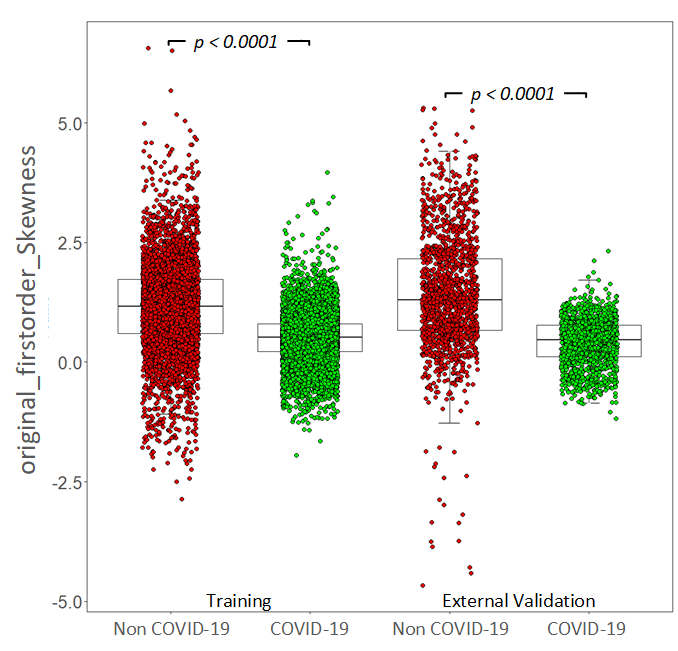


**Appendix D eFigure 3.** Distribution of values of the “original_firstorder_Skewness” feature between the COVID-19 images and non-COVID-19 images in the training and external validation datasets.


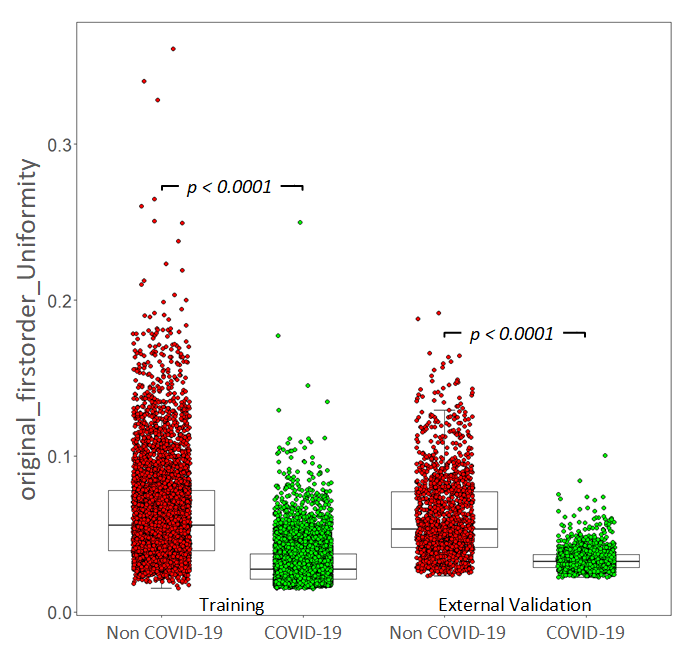


**Appendix D eFigure 4.** Distribution of values of the “original_firstorder_Uniformity” feature between the COVID-19 images and non-COVID-19 images in the training and external validation datasets.


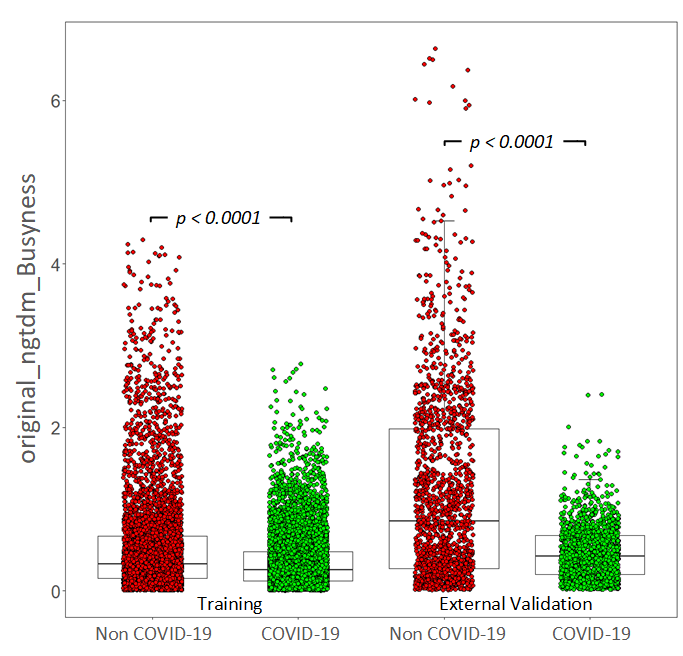


**Appendix D eFigure 5.** Distribution of values of the “original_ngtdm_Busyness” feature between the COVID-19 images and non-COVID-19 images in the training and external validation datasets.


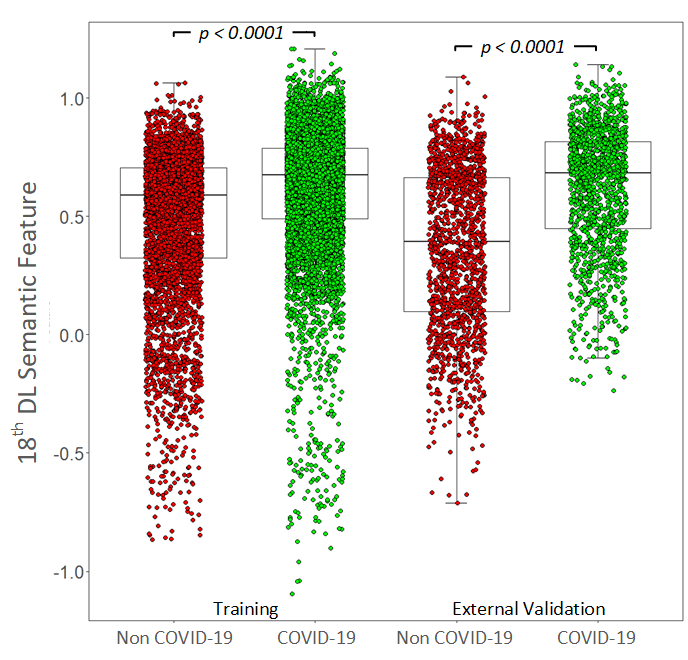


**Appendix D eFigure 6.** Distribution of values of the 18^th^ deep learning semantic feature between the COVID-19 images and non-COVID-19 images in the training and external validation datasets.


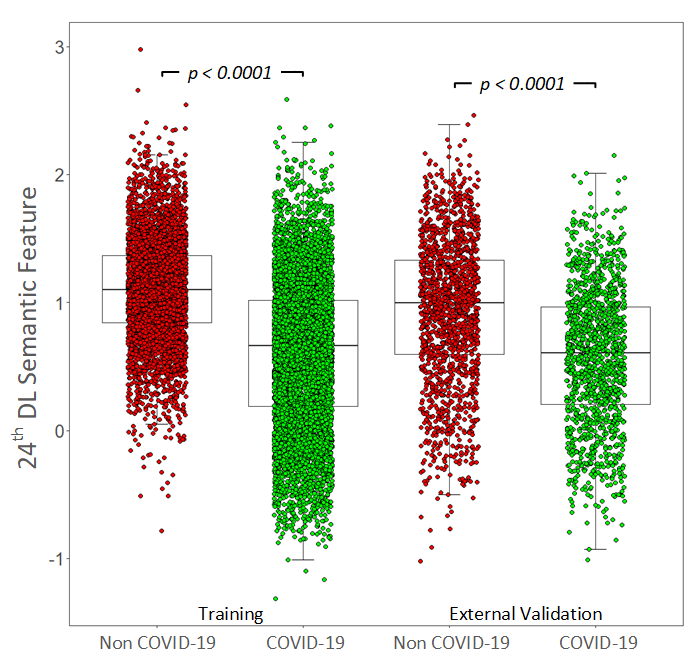


**Appendix D eFigure 7.** Distribution of values of the 24^th^ deep learning semantic feature between the COVID-19 images and non-COVID-19 images in the training and external validation datasets.


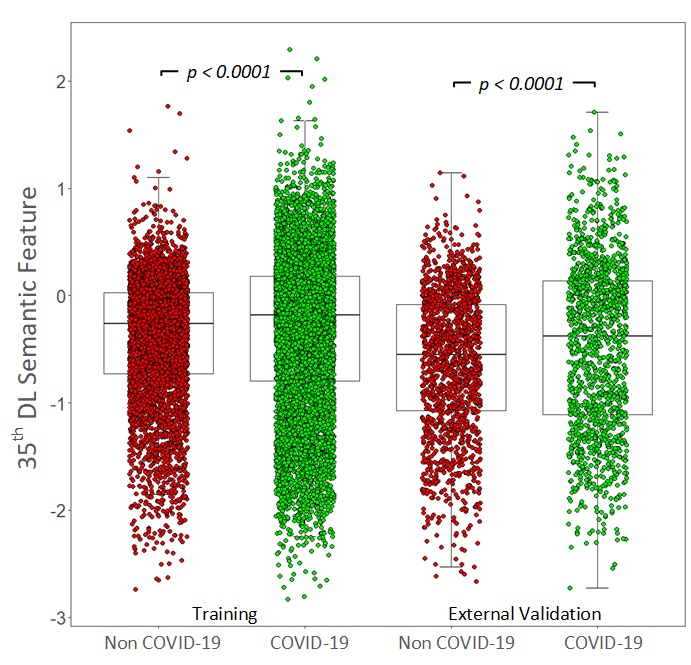


**Appendix D eFigure 8.** Distribution of values of the 35^th^ deep learning semantic feature between the COVID-19 images and non-COVID-19 images in the training and external validation datasets.


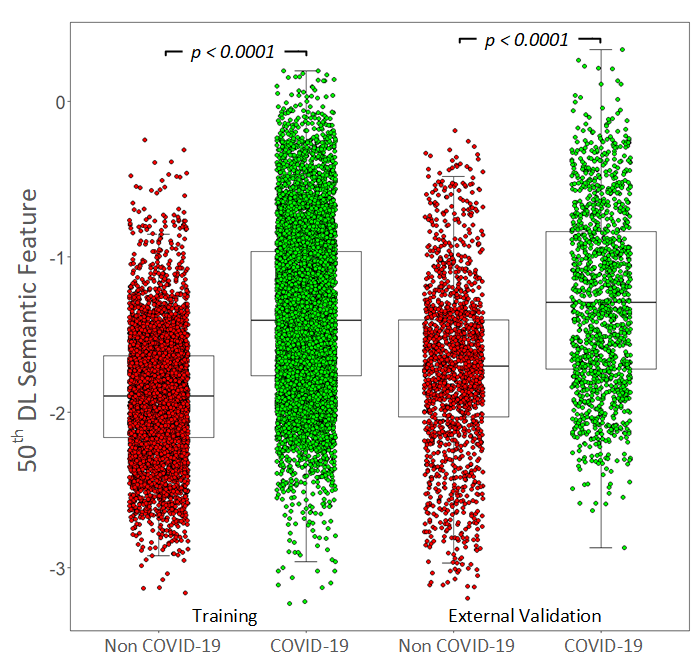


**Appendix D eFigure 9.** Distribution of values of the 50^th^ deep learning semantic feature between the COVID-19 images and non-COVID-19 images in the training and external validation datasets.


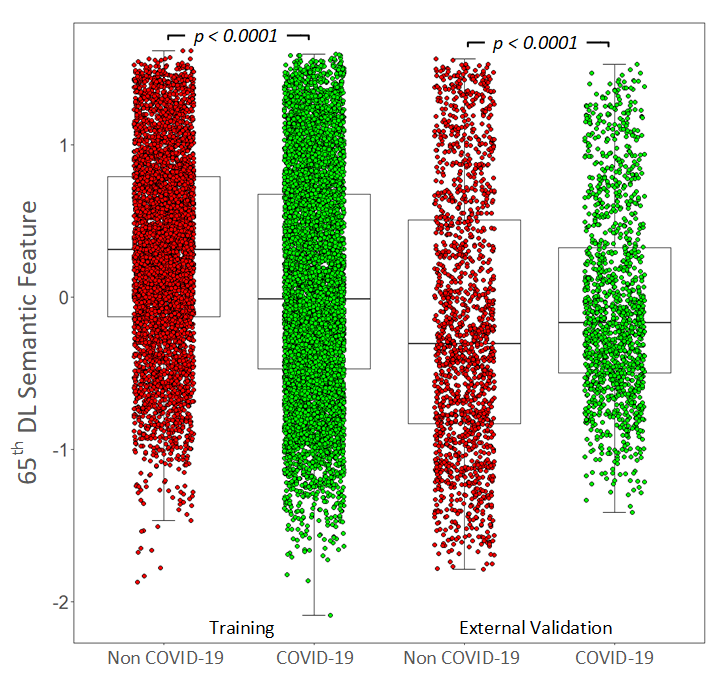


**Appendix D eFigure 10.** Distribution of values of the 65^th^ deep learning semantic feature between the COVID-19 images and non-COVID-19 images in the training and external validation datasets.


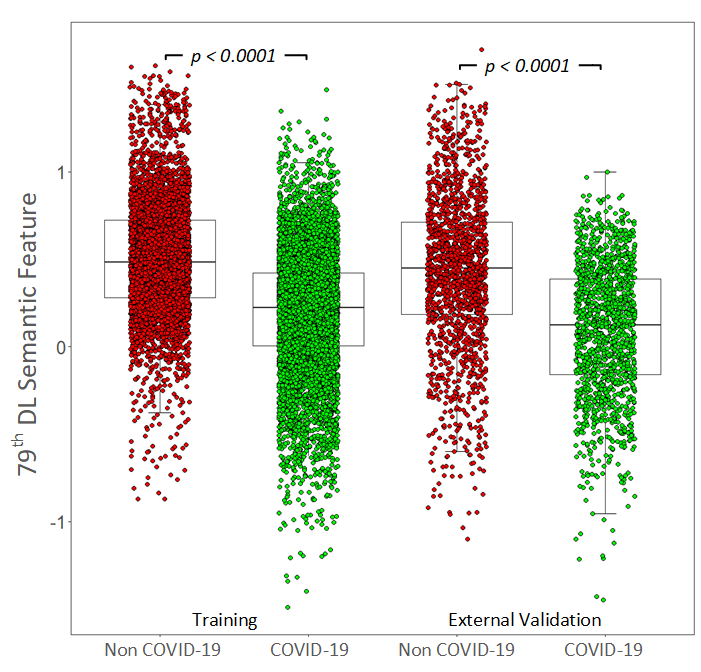


**Appendix D eFigure 11.** Distribution of values of the 79^th^ deep learning semantic feature between the COVID-19 images and non-COVID-19 images in the training and external validation datasets.
